# *Histoplasma* tympanomastoiditis: Case report and literature review

**DOI:** 10.1016/j.idcr.2023.e01774

**Published:** 2023-04-26

**Authors:** Terrence Park, Gordon Love, Victoria Burke

**Affiliations:** Louisiana State University Health Sciences Center Section of Infectious Diseases, 1542 Tulane Avenue, Suite 331C, New Orleans, LA 70112, USA

**Keywords:** Fungal otomastoiditis, Histoplasmosis

## Abstract

*Histoplasma* is a dimorphic fungus capable of producing a diverse array of clinical pathology in humans dependent upon the host immune status. Acute symptomatic infection typically presents as an isolated pulmonary or nodal disease in immunocompetent patients with extra-thoracic manifestations rarely seen in this population. In this report, we describe a novel case of *Histoplasma capsulatum* tympanomastoiditis in an immunocompetent patient who presented with progressively worsening purulent otorrhea, vertigo, and facial nerve palsy. He was successfully managed with surgical debridement and a prolonged antifungal course.

## Introduction

*Histoplasma* is a thermally dimorphic fungus that exists as a hyaline mold in the ambient natural environment and as a yeast at body temperature [1], [2]. It is ubiquitous to the Ohio and Mississippi River valleys in the continental United States as well as parts of Central America, South America, the Caribbean, Africa, India, Southeast Asia, and Australia [3]. Acute infection in the immunocompetent patient, when not completely asymptomatic, presents as either mediastinal adenitis or pneumonia that is often clinically indistinguishable from community acquired pneumonia [4]. In patients with severely compromised adaptive immunity due to HIV/AIDS, solid organ transplantation, or following receipt of chronic immunosuppressive therapy, dissemination can occur to distant sites including the lymph nodes, liver, spleen, bone marrow, mucocutaneous tissues, and gastrointestinal tract [1], [3], [4]. Mucocutaneous lesions are frequently reported in the literature as a manifestation of disseminated histoplasmosis but are less commonly described as a primary disease manifestation following occupational or recreational microconidia inoculation [1], [5], [6], [7], [8], [9]. Cutaneous lesions of the ear with associated otomastoiditis due to *Histoplasma* have not been described to date in the medical literature. In this report, we describe a novel case of unilateral tympanomastoiditis due to biopsy proven histoplasmosis in an immunocompetent patient.

## Case presentation

A 52-year-old man with a past medical history of hypertension, hyperlipidemia, type 2 diabetes, obstructive sleep apnea, long-term tobacco use, and a left tympanic membrane defect presented with several months of progressively worsening acute on chronic left ear drainage, vertigo, and facial droop. The patient reported intermittent serous left ear drainage since childhood following several episodes of otitis media that were ultimately complicated by abscess development with a subsequent tympanic membrane defect following drainage. He noted that over the preceding three months this drainage became increasingly sanguinopurulent and was accompanied by intermittent vertigo, hearing loss on the left, and pain radiating from his ear down the side of his neck. His symptoms failed to improve with outpatient treatment with a 10-day course of amoxicillin-clavulanate and prednisone. A week prior to presentation, he developed acute onset left facial droop, ultimately prompting him to seek medical care. He otherwise denied fevers, chills, sweats, unintentional weight loss, headache, vision changes, dysphagia or falls. Of note, his social and exposure history were notable for residence in a rural area of southern Louisiana where he tended to a quail and chicken coop on his property, harvesting eggs and clearing away bird guano on a regular basis.

Physical examination at presentation was notable for a temperature of 97.9°F and otherwise unremarkable vital signs. His neurologic examination revealed a left facial nerve palsy. No meningismus was noted. Otoscopic examination of the left ear was notable for a fleshy pink soft tissue mass emanating from the medial canal and thin sanguinopurulent discharge draining from a chronically perforated tympanic membrane. An audiogram revealed mixed conductive and sensorineural hearing loss on the left side.

**Fig. 1:**
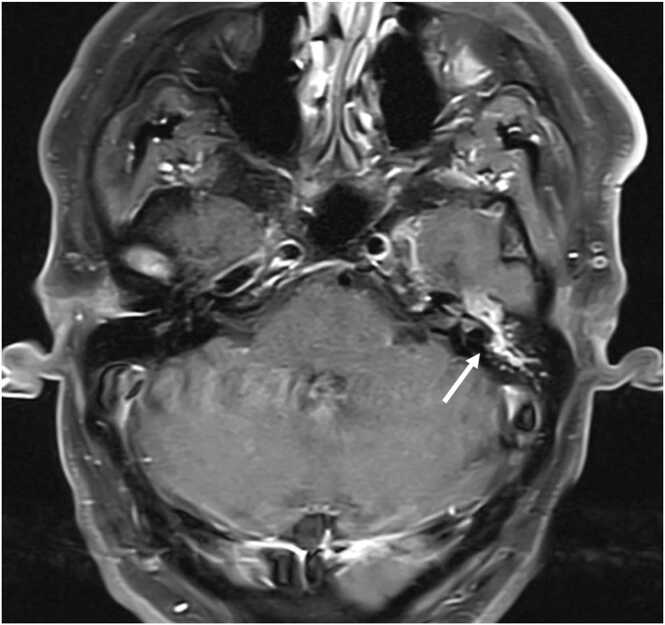
T1 fast spin echo brain magnetic resonance imaging showing soft tissue or fluid density material within the left middle ear cavity and left mastoid air cells as well as bony demineralization of the ossicular chain (as denoted by the white arrow).

Laboratory tests were notable for a white blood cell count of 5700 cells/μL (normal: 4000–11,000 cells/μL) and an unremarkable complete metabolic panel. Initial computed tomography scan of the head with thin cuts of the temporal bone was obtained that revealed opacification of the left middle ear and mastoid sinuses and erosive changes of the ossicular chain and adjacent temporal bone. Subsequent magnetic resonance imaging of the brain confirmed the presence of a left ear canal soft tissue mass with associated tympanomastoid opacification (Fig. 1).

**Fig. 2:**
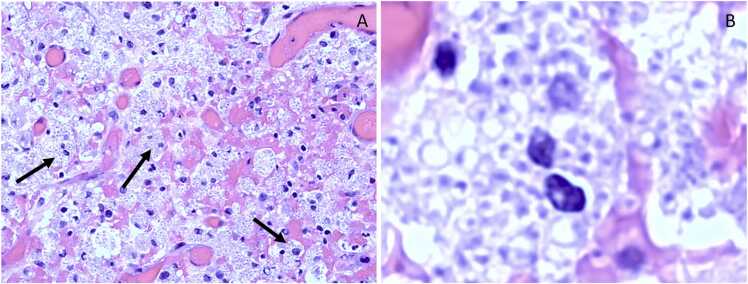
Small, ovoid, narrow-based budding yeast imbedded within either monocytes or macrophages (as depicted by the arrows in Fig. 2A and on Fig. 2B).

**Fig. 3:**
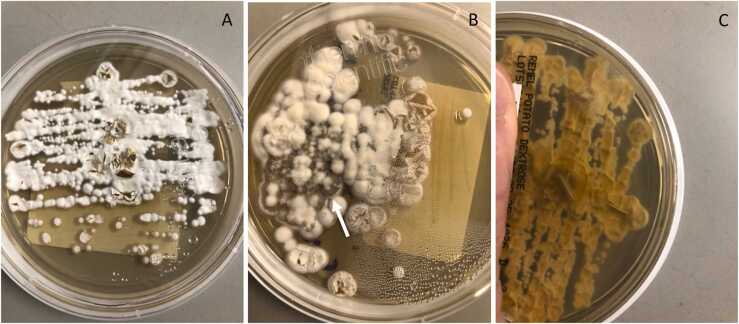
Potato dextrose agar showing growth of *Histoplasma capsulatum* after several weeks. Characteristic white cotton-like colonies can readily be seen (Fig. 3A). With the passage of time, transitions in color can occur from a whitish appearance (Fig. 3B) to a gray to brownish appearance (Fig. 3C).

Initial concern per otolaryngology was for an obstructive cholesteatoma as the cause of his symptoms. He subsequently underwent left radical tympanomastoidectomy, meatoplasty, facial nerve decompression, and full thickness skin graft for canal reconstruction with significant improvement in his symptoms post-operatively. He was initially started on broad empiric antibiotic coverage by his primary otolaryngology team when formal histopathology returned, surprisingly revealing numerous intracellular yeasts with invasion into soft tissue and ossicular bones, consistent with histoplasmosis (Fig. 2). At this time, the infectious diseases consult team was contacted and his empiric antimicrobials were tailored to also include liposomal amphotericin 3 mg/kg intravenously every 24 h. Operative tissue cultures also grew a variety of anaerobic flora *(Fusobacterium varium, Anaerococcus* species, *Parvimonas micra,* and *Prevotella* species)*,* prompting tailoring of his broad bacterial coverage to ampicillin-sulbactam 3 g intravenously every 6 h for concurrent bacterial superinfection. After several weeks, *Histoplasma capsulatum* ultimately grew from operative tissue cultures as well to further confirm the primary underlying diagnosis of *Histoplasma* otomastoiditis (Fig. 3).

Given the rarity of extrapulmonary histoplasmosis in the absence of disseminated disease, he underwent additional evaluation that failed to show evidence of immunocompromise or disseminated infection, including unremarkable urine *Histoplasma* antigen testing, HIV testing, lymphocyte subsets, immunoglobulin subsets, and computed tomography scan of the chest, abdomen, and pelvis. A lumbar puncture was also performed at the advent of antifungal therapy that excluded contiguous central nervous system seeding.

After dissemination was excluded and following significant clinical improvement with a week of liposomal amphotericin, his antifungal therapy was transitioned to maintenance itraconazole solution 200 mg by mouth every 12 h (following a 3-day dosage load). He subsequently completed a 6-week course of ampicillin-sulbactam and a 3-month course of itraconazole for his polymicrobial tympanomastoiditis with possible residual skull osteomyelitis after debridement. He has been seen in clinic by both infectious diseases and otolaryngology following conclusion of this therapy with lasting clinical improvement of his symptoms excluding some residual sensorineural hearing loss in the affected ear.

## Discussion

Fungal otomastoiditis, or fungal infection of the middle ear and mastoid air cells, is a rare entity that most commonly occurs in immunocompromised patients, including persons living with HIV/AIDS or hematologic malignancies [10]. Patients typically present with chronic intermittent otorrhea, otalgia, vertigo, and often cranial nerve palsies that persist despite repeated courses of antibiotics and steroids [11]. Fungal organisms are thought to gain access to the middle ear and its associated structures through one of three major routes: tympanogenic spread via membrane defect, contiguous spread from infection in an adjacent meningeal or nasopharyngeal site, or hematogenous spread [10]. Previous case reports have described numerous fungal species as causative agents, including various *Aspergillus, Mucorales,* and *Scedosporium* [10]. Notably, dimorphic fungi have been implicated infrequently as the etiologic agent of otomastoiditis, with only a few cases attributed to *Blastomyces dermatiditis* previously described in the medical literature [12], [13]. To our knowledge, this report describes the first documented case of otomastoiditis due to *Histoplasma capsulatum*.

*Histoplasma* is a thermally dimorphic fungus that thrives in soil enriched with bird and bat excrement, which is postulated to accelerate organism sporulation [3], [4]. Occupational and recreational activities which disturb this organism-laden soil – including construction, agriculture, forestry, spelunking, hunting, and bird keeping – predispose to histoplasmosis through aerosolization of microconidia [1], [3], [4]. Although the usual portal of entry for infection is pulmonary via inhalation of microconidia, cases of localized disease due to organism entry through tissue defects have been described in the setting of high burden occupational and recreational exposures [1], [5], [6], [7], [8], [9]. In the immunocompetent patient described in our case, it is presumed that the patient was exposed to a high burden of *Histoplasma* microconidia while tending his quail and chicken coop, with the organism gaining access to his middle ear and mastoids through his tympanic membrane defect.

Localized histoplasmosis, including that of otomastoiditis described in our case, is typically first suggested by histopatholology findings that demonstrate small (2–4 µm), ovoid, narrow-based budding yeasts within macrophages or monocytes [2]. Specific fungal stains including both Grocott-Gomori Methanamine-Silver (GMS) and periodic acid-Schiff (PAS) stains best highlight the yeast form in tissues [2], [4]. In immunocompetent patients, the organisms can often be visualized within both necrotizing and non-necrotizing granulomas [2], [4]. Microbiologic confirmation of infection is often slower than histopathology given that the organism often requires up to 4 weeks for growth on dedicated enriched fungal media, such as potato dextrose agar [2], [4].

Given the slow growth of the organism from culture, molecular methods have been developed to aid in acute diagnosis, including both *Histoplasma* antigens and antibodies. Urinary antigen testing has a reported sensitivity of 91.8% for acute disseminated infection in immunocompromised hosts but unfortunately is less sensitive in the detection of localized disease in immunocompetent hosts, including our unique case of otomastoiditis [4], [14]. Antibody testing by immunodiffusion or complement fixation in contrast has improved sensitivity for the diagnosis of acute infection in immunocompetent hosts but often requires several weeks for convalescent positivity and can remain positive for years after infection, confounding its utility as an acute diagnostic [2], [4], [14].

Treatment of histoplasmosis is typically dependent upon the severity of illness and host immune status, with mandatory treatment only recommended for progressive disseminated infection, significant pulmonary disease, or other concerning manifestations, particularly in immunocompromised hosts [15]. An initial induction course of liposomal amphotericin is typically given for severe manifestations with a transition to oral itraconazole when clinical improvement or stabilization has been noted [4], [15]. In the case of our patient, we elected to administer an initial one-week course of induction liposomal amphotericin after surgical debridement prior to transitioning to a prolonged course of itraconazole solution based on the severity of our patient’s initial presentation with cranial nerve deficits. Consistent with other described cases of fungal otomastoiditis, our patient was managed with a combination of surgical debridement and antifungal agents with significant clinical improvement [10], [11].

## Conclusion

Although fungal otomastoiditis is a rare condition, it is an important etiology to consider as the cause of persistent otorrhea, otalgia, vertigo, and cranial nerve palsies despite prior antibiotics courses, particularly in immunocompromised patients or those with tympanic membrane defects [10], [11]. Obtainment of appropriate tissue samples for culture and histopathology in these refractory cases is critical in the identification of unusual pathogens, including *Histoplasma capsulatum* as reported in our case. Once diagnosed, this entity is best managed with a combined surgical and medical approach including debridement with concurrent antifungal therapy [15].

## Ethical approval

Obtained

## Consent

Written informed consent to write this case report was obtained from the patient.

## Funding

This research did not receive any specific grant from funding agencies in the public, commercial or not-for-profit sectors.

## Declaration of Competing Interest

None.
